# Factors related to the provision of home-based end-of-life care among home-care nursing, home help, and care management agencies in Japan

**DOI:** 10.1186/s13104-015-1418-z

**Published:** 2015-09-12

**Authors:** Ayumi Igarashi, Takeshi Kurinobu, Ayako Ko, Yuko Okamoto, Shino Matsuura, Mei Feng, Noriko Yamamoto-Mitani

**Affiliations:** School of Health Sciences and Nursing, Graduate School of Medicine, The University of Tokyo, 7-3-1 Hongo, Bunkyo-ku, Tokyo, 113-0033 Japan; Tokyo Metropolitan Institute of Gerontology, 35-2 Sakae-cho, Itabashi-ku, Tokyo 173-0015 Japan; Graduate School of Health Care Sciences, Tokyo Medical and Dental University, 1-5-45 Yushima, Bunkyo-ku, Tokyo, 113-8519 Japan; Faculty of Nursing, Japanese Red Cross College of Nursing, 4-1-3 Hiroo, Shibuya-ku, Tokyo, 150-0012 Japan; Department of Nursing, Faculty of Human Sciences, Sophia University, 7-1 Kioicho, Chiyoda-ku, Tokyo, 102-8554 Japan; Intensive Care Unit, West China Hospital of Sichuan University, No. 37 Guo Xue Xiang, Chengdu, Sichuan 610041 China

**Keywords:** Care management, Home-care nursing, Home help, End-of-life care, Long-term care, Community

## Abstract

**Background:**

To promote home death, it is necessary to clarify the institutional barriers to conducting end-of-life (EOL) care and consider strategies to deal with this process. This study aims to clarify institution-related factors associated with the provision of home-based EOL care cases, and to compare them among three different types of home-care agencies.

**Methods:**

We administered a cross-sectional survey throughout Japan to investigate the number and characteristics of EOL cases of home-care nursing (HN), home-help (HH) and care management (CM) agencies. Bivariate and multivariate analyses were performed for each type of agency to examine factors related to the provision of EOL care.

**Results:**

378 HN agencies, 274 HH agencies, and 452 CM agencies responded to the distributed questionnaire. HN agencies had on average 2.1 (SD = 4.0; range 0–60) home-based EOL cases in the last 3 months, while HH agencies had 0.9 (SD = 1.3; range 0–7) and CM agencies had 1.5 (SD = 2.2; range 0–18) in the last 6 months. In a multivariable analysis of HN agencies, a large number of staff (OR: 1.52; p < 0.001) and a large number of collaborating CM agencies (OR: 1.08; p = 0.008) were positively associated with the provision of EOL care; in HH agencies, accepting EOL clients in the agency (OR: 3.29; p < 0.001) was positively associated with the provision of EOL care; in CM agencies, the number of staff (OR: 1.21; p = 0.037), the number of collaborating HH agencies (OR: 1.07; p = 0.032), and whether home-care nurses and home helpers visit clients together (OR: 1.89; p = 0.007) were positively associated with the provision of EOL care.

**Conclusion:**

The agency’s size and the inter-agency collaborative system seemed most important among HN agencies and CM agencies, while institutional preparedness for EOL was most important for HH agencies. These findings represent important new information for targeting different effective strategies in the promotion of home-based EOL care, depending on the agency type.

## Background

In response to the unprecedented increase in the older population demographic, the Japanese government has begun establishing a “community comprehensive care system” in which the principle of “aging-in-place”—being able to continue living in one’s own home adapting to changing needs and conditions [1]—and associated values such as home-based end-of-life (EOL) care should be fully practiced [2]. Home-based EOL care is also preferred by the Japanese, as research has shown that forty to fifty percent of the public, as well as those suffering from cancer, prefer to stay at home until the end of their lives [3, 4]. However, in 2012, only 13 % (161,242 out of 1,256,359 deaths) of people in Japan died at home [5]. To promote home death, it is necessary to clarify the factors related to conducting EOL care and to consider effective strategies to deal with the process.

Home-based EOL care in Japan is managed by the long-term care insurance (LTCI) system in the sense that essential services for EOL care, namely home-care nursing (HN), home-help (HH), and care management (CM) services, are provided through the LTCI system. In the LTCI system, however, there are multiple barriers for those services to effectively support home-based EOL. First of all, since home-care agencies in Japan are generally managed on a small scale with a limited number of staff [6], it is not often possible to respond to urgent situations, which is critical for EOL care. Second, there is inter- and intra-professional variability among staff members in terms of their preparedness and training to provide EOL care. Many home helpers and care managers do not have similar opportunities, while nurses generally gain experience in EOL care through previous work in hospitals, as shown in the statistics that almost all (99.6 % in 2014) of new graduate nurses gain employment in a hospital [7]. Third, the type of inter-professional work done in the community is substantially different from what is done in hospitals and no model of integration or learning has been made available. Community-based inter-professional work involves a variety of professionals including social as well as medical care workers, profit, non-profit and public sectors that have very different philosophies and administrative policies. As a result, there could be psychological and physical barriers between them [8, 9]. In order to promote home-based EOL care, it is necessary to carefully examine the factors that enable or hinder EOL care in each type of agency and develop effective strategies for supporting them.

Patient-related and provider-related factors of home-based EOL care have been revealed in previous studies: patients’ low functional status [10, 11], living with relatives [10], patients and/or family preference [12–14], home care and its intensity [10], the frequency of physicians’ home visits [15], and affiliation of the attending physician [13]. Meanwhile, it has not been clear which characteristics of home-care agencies were associated with the possibility of providing EOL care, nor whether there is a difference in the associated characteristics among different types of home-care agencies.

This study aimed to clarify institutional factors associated with the provision of home-based EOL care, and to compare them among three different types of home-care agencies. In this study, we focused on HN, HH, and CM agencies, as they have important roles in home-based EOL care in the LTCI system.

### Long-term care insurance in Japan

Japan’s LTCI system was introduced in 2000 [16] in response to the ongoing rise of the aging population. Under the LTCI system, those who are 65 years and over, or those who are 40–64 years of age with specific age-related diseases, are eligible to receive social and nursing care within a fixed budget based on their level of care need certification. The necessary LTCI services for each older adult are determined by a comprehensive assessment by a care manager, newly introduced in the LTCI system, who belongs to a CM agency. The care managers are certified by prefectural governments and come from a variety of professional backgrounds, including care workers, social workers, and nurses.

Under the management of a care manager, home-care nurses provide home-care nursing services (e.g., management of chronic illness, care for daily life, and medical procedure) in HN agency; home helpers provide home-help services (physical care and livelihood support) in a HH agency. While HN, HH, and CM services are all provided under the LTCI system, the services themselves are provided by separate agencies, even if they are affiliated with the same corporation. For instance, home helpers are employed and trained by HH agencies, just as home-care nurses are employed by HN agencies.

## Methods

### Participants and study design

A cross-sectional survey was conducted from October to November 2011 by mailing self-administered, anonymous questionnaires to HN, HH, and CM agencies. By using systematic sampling, we selected 1000 out of 5198 HN agencies, 1000 out of 26,026 HH agencies, and 1200 out of 30,548 CM agencies. In the sampling, the participating agencies were selected at regular intervals from the agency’s lists, which were provided by Welfare and Medical Service NET work System (WAM NET), a healthcare information network system in Japan. Agencies were excluded in those municipalities covered by the Disaster Relief Act, i.e. those areas affected by the Tohoku Region Pacific Coast Earthquake and Northern Nagano Prefecture Earthquake in March 2011.

In the survey, we asked one selected home-care nurse, home helper, or care manager at each agency to answer the questionnaire and return it to one of the authors. A reminder was also mailed before the deadline. Returning the completed questionnaire was deemed as consent to participate in the study. The research protocol was examined by the ethics committee of the Japan Visiting Nursing Foundation.

### Measurement

In this study, data pertaining to agency characteristics and the provision of EOL care in each agency was collected.

#### Number of end-of-life cases

The number of EOL cases in the past 3 (HN agencies) or 6 (HH and CM agencies) months was requested. We determined the 3/6 months time frame based on estimating the differences in the numbers and distribution of EOL cases between HN agencies and HH/CM agencies. This disparity is due to the differences of purposes of providing the services: while home-care nursing services are introduced for EOL care (the National Association for Home-visit Nursing Care), HH and CM services are not introduced for this purpose.

#### Agency characteristics

Agency characteristics included in the questionnaire consisted of the following aspects: (1) the number of professional staff (as per full time equivalent: FTE), (2) the presence of staff possessing a nursing license (only in CM agency), (3) the number of clients in the previous month, (4) agency ownership, (5) the presence of other home-care agencies in the same organization, (6) the number of collaborating agencies (i.e., healthcare facilities including hospitals and clinics, CM agencies, HN agencies, HH agencies), (7) whether the agency was certified for additional reimbursement for intensive services, (8) whether the agency actively accepted EOL cases (only in HH agencies), and (9) whether home-care nurses and home helpers could visit clients together in the region.

In the LTCI and healthcare insurance systems, home-care agencies can gain additional reimbursement if they provided care for clients with specific situations. HN agencies could gain several types of reimbursement from the LTCI and healthcare insurance systems by providing round-the-clock care, EOL care, care for clients who need medical treatment, and so on. On the other hand, HH and CM agencies can gain additional reimbursement from the LTCI when they have a large number of certified staff and provide care for clients who have severe care need levels [17]. In this study, we defined an agency as being certified for “additional reimbursement for intensive services” based on the following aspects: whether HN agencies gained any reimbursement from the healthcare insurance and LTCI systems, and whether HH or CM agencies gained reimbursement that requires them to provide care to clients with severe care need levels.

### Data analyses

Following the analyses of the descriptive statistics of each variable, we divided the number of EOL cases into two categories: whether an agency provided EOL care (=1) or not (=0). Afterwards, bivariate analyses were conducted in order to identify factors associated with the provision of EOL care by conducting a Mann–Whitney U test or Chi square tests, depending on the nature of the independent variables.

After the bivariate analyses, three models were developed and tested for each type of agency to explore the possible significant factors that were most relevant to the provision of EOL care. The variables associated with the provision of EOL care at *p* < .20 in the bivariate analyses were inputted into the logistic regression analysis.

The analyses were performed using the statistical package SAS for Windows, version 9.3 (SAS Institute, Cary, NC, USA). The significance level was set at less than 0.05 (two-tailed).

## Results

Of the distributed questionnaires, 11, 26, and 21 were returned from HN, HH, and CM agencies, respectively, due to an unknown or incorrect address; 378 (38.2 %) home-care nurses, 305 (26.0 %) home helpers, and 476 (40.4 %) care managers returned the questionnaires. Due to missing data, information concerning the number of clients who died at home was only present in the questionnaires from 371 (37.5 %) HN agencies, 274 (23.3 %) HH agencies, and 452 (38.3 %) CM agencies. These questionnaires were used for the analyses that will follow.

### Agency characteristics and the provision of EOL care (Table 1)

First, we examined the descriptive statistics of the variables. The median numbers of professional staff were 4.0 for HN agencies, 6.1 for HH agencies, and 2.0 for CM agencies; the number of clients per month was largest for CM agencies, with median numbers of 58.0. While HN agencies collaborated with a median of 10.0 CM agencies, CM agencies only collaborated with a median of 2.0 HN agencies. While a large percentage of HN agencies gained additional reimbursement for long-term care insurance (96.5 %) or healthcare insurance (89.3 %), only a few HH agencies (6.4 %) and CM agencies (3.2 %) gained the additional reimbursement, despite somewhat different reimbursement requirements for the three types of agencies.

**Table 1:** Characteristics of homecare nurse, home helper, and care manager agencies

	Homecare nurse	Home helper	Care manager
	n = 371	n = 274	n = 452
	n (%)	n (%)	n (%)
	Median (25–75 percentile)	Median (25–75 percentile)	Median (25–75 percentile)
Number of staff (FTE)	4.0 (3.0–5.8)	6.1 (3.4–11.6)	2.0 (1.35–3.45)
Nursing staff			183 (40.5)
Number of clients/1 month	45.0 (28.0–75.0)	42.0 (25.0–73.0)	58.0 (32.0–97.0)
Agency ownership			
Social welfare corporation	39 (10.6)	72 (26.4)	133 (29.6)
Profit corporation	102 (27.7)	144 (52.7)	166 (37.0)
Healthcare corporation	136 (37.0)	29 (10.6)	84 (18.7)
Others	91 (24.7)	28 (10.3)	66 (14.7)
Other healthcare facility owned by the same organization			
Care management agency	235 (63.3)	196 (76.6)	
Homecare nursing agency		45 (17.6)	70 (17.7)
Home help agency	115 (31.0)		201 (50.9)
Healthcare facility	97 (26.1)	13 (5.1)	52 (13.2)
Non-bed clinic	44 (11.9)	10 (3.9)	22 (5.6)
Number of collaborating HN agencies	–	–	2.0 (1.0–3.0)
Number of collaborating HH agencies	–	–	5.0 (3.0–9.0)
Number of collaborating CM agencies	10.0 (5.0–18.0)	6.0 (3.0–10.0)	–
Number of collaborating healthcare facilities	13.0 (8.0–25.0)	–	–
Additional reimbursement for intensive service			
By long-term care insurance	354 (96.5)	17 (6.4)	14 (3.2)
By healthcare insurance	326 (89.3)	–	–
Accepting end-of-life clients	–	141 (52.0)	–
Homecare nurses and home helpers could visit clients together	264 (73.3)	155 (58.1)	266 (61.1)
Number of clients who died at home/3 or 6 months, mean ± SD	2.1 ± 4.0	0.9 ± 1.3	1.5 ± 2.2

The figures of each item were not equal to the total number of the participants due to missing values; percentages for each item were calculated after excluding missing values

*SD* standard deviation

HN agencies had on average 2.1 (SD = 4.0; range 0–60) home-based EOL cases in the last 3 months, while HH agencies had 0.9 (SD = 1.3; range 0–7) and CM agencies had 1.5 (SD = 2.2; range 0–18) in the last 6 months.

### Bivariate analysis (Table 2)

Second, bivariate analyses were conducted to discover associations between the provision of EOL care cases and certain factors. For HN agencies, the following variables were positively associated with the provision of EOL care in the last 3 months at p < 0.2: the number of FTE staff (p < 0.001), the number of clients/month (p < 0.001), the type of agency corporation (p = 0.079), the number of collaborating CM agencies (p < 0.001), the number of collaborating healthcare facilities (p < 0.001), additional reimbursement for intensive care by the LTCI (p = 0.001) and the healthcare insurance systems (p < 0.001), and whether home-care nurses and home helpers could visit clients together (p = 0.067).

**Table 2:** Associations between the provision of EOL care and agency characteristics

	Homecare nurse, n = 371			Home helper, n = 274			Care manager, n = 452		
	Provision of EOL Care			Provision of EOL Care			Provision of EOL Care		
	No (n = 140)	Yes (n = 231)	p-value	No (n = 152)	Yes (n = 122)	p-value	No (n = 198)	Yes (n = 54)	p-value
Agency characteristics									
Number of staff (FTE)	3.1 (2.6–4.0)	4.5 (3.2–6.5)	<0.001^a^	4.8 (3.0–9.7)	8.4 (4.0–14.6)	0.001^a^	2.0 (1.0–3.0)	2.6 (2.0–4.0)	<0.001^a^
Nursing staff									
Yes							70 (35.4)	114 (44.7)	0.044^b^
No							128 (64.7)	141 (55.3)	
Number of clients/1 month	36.0 (20.0–56.0)	52.0 (34.0–88.0)	<0.001^a^	35.0 (21.0–64.0)	53.0 (31.0–80.0)	0.001^a^	42.0 (23.0–74.0)	68.5 (39.0–108.5)	<0.001^a^
Agency ownership									
Social welfare corporation	19 (13.8)	20 (8.7)	0.079^b^	42 (27.6)	30 (24.8)	0.574^b^	53 (26.8)	80 (31.8)	0.303^b^
Profit corporation	38 (27.5)	64 (27.8)		77 (50.7)	67 (55.4)		81(40.9)	85 (33.7)	
Healthcare corporation	56 (40.6)	80 (34.8)		19 (12.5)	10 (8.3)		33 (16.7)	52 (20.6)	
Others	25 (18.1)	66 (28.7)		14 (9.2)	14 (11.6)		31 (15.7)	35 (13.9)	
Other healthcare faclity owned by the same organization									
Care management agency									
Yes	84 (60.0)	151 (65.4)	0.298^b^	101 (72.7)	95 (81.2)	0.108^b^			
No	56 (40.0)	80 (34.6)		38 (27.3)	22 (18.8)				
Homecare nursing agency									
Yes				21 (15.1)	24 (20.5)	0.258^b^	24 (13.7)	47 (21.3)	0.052^b^
No				118 (84.9)	93 (79.5)		151 (86.3)	174 (78.7)	
Home help agency									
Yes	43 (30.7)	72 (31.2)	0.927^b^				86 (49.1)	115 (52.0)	0.567^b^
No	97 (69.3)	159 (68.8)					89 (50.9)	106 (48.0)	
Hospital									
Yes	36 (25.7)	61 (26.4)	0.883^b^	8 (5.8)	5 (4.3)	0.591^b^	23 (13.1)	30 (13.6)	0.900^b^
No	104 (74.3)	170 (73.6)		131 (94.2)	112 (95.7)		152 (86.9)	191 (86.4)	
Non-bed clinic									
Yes	19 (13.6)	25 (10.8)	0.427^b^	6 (4.3)	4 (3.4)	0.712^b^	7 (4.0)	15 (6.8)	0.229^b^
No	121 (86.4)	206 (89.2)		133 (95.7)	113 (96.6)		168 (96.0)	206 (93.2)	
Number of collaborating HN agencies							2.0 (0.0–3.0)	2.0 (1.0–4.0)	<0.001^a^
Number of collaborating HH agencies							4.0 (2.0–7.0)	6.0 (4.0–10.0)	<0.001^a^
Number of collaborating CM agencies	7.0 (3.0–12.0)	13.0 (7.0–20.0)	<0.001^a^	6.0 (3.0–10.0)	6.0 (4.0–12.0)	0.105^a^			
Number of collaborating healthcare faclities	11.0 (6.0–20.0)	16.0 (9.0–28.0)	<0.001^a^						
Additional reimbursement for intensive service									
By LTCI									
Yes	127 (92.0)	227 (99.1)	<0.001^b^	29 (19.7)	36 (30.8)	0.039^b^	3 (1.6)	11 (4.5)	0.087^b^
No	11 (8.0)	2 (0.9)		118 (80.3)	81 (69.2)		189 (98.4)	236 (95.6)	
By healthcare insurance									
Yes	109 (80.7)	217 (94.4)	<0.001^b^						
No	26 (19.3)	13 (5.7)							
Accepting end-of-life clients				59 (39.1)	82 (68.3)	<0.001^b^			
				92 (60.9)	38 (31.7)				
Homecare nurse and home helper could visit client together									
Yes	93 (67.9)	171 (76.7)	0.067^b^	86 (58.9)	69 (57.0)	0.757^b^	97 (52.7)	170 (67.5)	0.002^b^
No	44 (32.1)	52 (23.3)		60 (41.1)	52 (43.0)		87 (47.3)	82 (32.5)	

The figures of each item were not equal to the total number of the participants due to missing values; percentages for each item were calculated after excluding missing values

^a^Mann–Whitney U test

^b^Chi square tests

For HH agencies, the following variables were positively associated with the provision of EOL care at p < 0.2: the number of FTE staff (p = 0.001), the number of clients/month (p = 0.001), owning a CM agency in the same organization (p = 0.108), the number of collaborating CM agencies (p = 0.105), additional reimbursement for intensive service by LTCI (p = 0.039), and acceptance of EOL clients (p < 0.001).

For CM agencies, variables positively associated with the provision of EOL care at p < 0.2 were as follows: the number of FTE staff (p < 0.001), care manager(s) who possess(es) nursing license (p = 0.044), the number of clients/month (p < 0.001), owning an HN agency in the same organization (p = 0.052), the number of collaborating HN agencies (p < 0.001), the number of collaborating HH agencies (p < 0.001), additional reimbursement for intensive service (p = 0.087), and whether home-care nurses and home helpers could visit clients together (p = 0.002).

For HN agencies and CM agencies, the number of clients/month were strongly correlated with the number of FTE in Spearman’s rank-correlation coefficient (r = 0.758 and 0.900, respectively), but for HH agencies, the correlation was not strong (r = 0.568). Furthermore, in all types of agencies, the correlation with the number of FTE and other variables were not strong (r < 0.5). Therefore, we excluded the number of clients for HN and CM agencies as an independent variable in the subsequent analyses.

### Multivariate analysis (Table 3)

Lastly, we conducted logistic regression analyses to identify factors related to the provision of EOL care cases of each agency. In the analysis of HN agencies, the large number of FTE staff (OR: 1.52; 95 % CI: 1.23–1.89; p < 0.001) and the large number of collaborating CM agencies (OR: 1.08; 95 % CI: 1.02–1.13; p = 0.008) were positively associated with the provision of EOL care in the last 3 months.

**Table 3:** Factors to the provision of EOL care

	Β	SE	OR	95 % CI		*p* value
Homecare nursing agency (n = 273)^a^						
Number of staff (FTE)	0.42	0.11	1.52	1.23	1.89	<.0001
Number of collaborating CM agencies	0.07	0.03	1.08	1.02	1.13	.008
Number of collaborating healthcare faclities	−0.03	0.02	0.97	0.93	1.01	.106
Agency ownership						
Social welfare corporation	(ref.)					
Profit corporation	0.44	0.25	2.60	0.96	7.02	.072
Healthcare corporation	−0.19	0.22	1.38	0.53	3.58	.391
Others	0.26	0.26	2.15	0.78	5.96	.317
Additional reimbursement for intensive service						
By LTCI	0.75	0.46	4.45	0.73	27.12	.105
By healthcare insurance	0.12	0.26	1.26	0.46	3.48	.656
Home-care nurse and home helper could visit client together	0.11	0.32	1.11	0.59	2.08	.743
Home help agency (n = 202)^b^						
Number of staff (FTE)	0.01	0.02	1.01	0.96	1.06	.781
Number of clients	0.003	0.005	1.00	0.99	1.01	.562
Other healthcare facility owned by the same organization	0.47	0.39	1.60	0.75	3.44	.224
Care management agency						
Number of collaborating CM agencies	0.02	0.03	1.02	0.96	1.08	.527
Additional reimbursement for intensive service	1.07	0.59	2.91	0.92	9.23	.069
Accepting end-of-life clients	0.60	0.16	3.29	1.79	6.05	<.0001
Care management agency (n = 352)^c^						
Number of staff (FTE)	0.19	0.09	1.21	1.01	1.45	.037
Nursing staff	0.29	0.25	1.33	0.82	2.17	.251
Number of collaborating HN agencies	0.03	0.07	1.03	0.91	1.18	.617
Number of collaborating HH agencies	0.06	0.03	1.07	1.01	1.13	.032
Other healthcare facility owned by the same organization						
Home-care nursing agency	0.35	0.33	1.42	0.74	2.71	.289
Additional reimbursement for intensive service	0.51	0.73	1.66	0.40	6.90	.486
Home-care nurse and home helper could visit client together	0.64	0.24	1.89	1.19	3.00	.007

^a^Hosmer–Lemeshow test: χ^2^ = 5.021, p = 0.755

^b^Hosmer–Lemeshow test: χ^2^ = 14.252, p = 0.075

^c^Hosmer–Lemeshow test: χ^2^ = 6.759, p = 0.563

In the analysis of HH agencies, accepting EOL clients in the agency (OR: 3.29; 95 % CI: 1.79–6.05; p < 0.001) was positively associated with the provision of EOL care in the last 6 months.

In the analysis of CM agencies, the following factors were positively associated with the provision of EOL care in the last 6 months: the number of FTE staff (OR: 1.21; 95 %CI: 1.01–1.45; p = 0.037), the number of collaborating HH agencies (OR: 1.07; 95 % CI: 1.01–1.13; p = 0.032), and whether home-care nurses and home helpers could visit clients together (OR: 1.89; 95 % CI: 1.19–3.00; p = 0.007).

## Discussion

This study compared factors influencing the provision of EOL care among three types of home-care agencies: HN, HH, and CM agencies. To the best of our knowledge, this is the first study to clarify agency-level institutional factors related to the provision of EOL care in communities. The results showed that factors such as the number of staff, acceptance of EOL cases, the number of collaborating agencies, and whether home-care nurses and home helpers could visit clients together were significantly associated with EOL cases in each agency. Differences in the significance of factors were found between the three types of agencies. We could consider effective approaches to promote higher quality home-based EOL care by considering these factors.

First, our findings indicated the importance of organizational support for home-based EOL care. It was found that the number of staff associated with EOL care cases in HN agencies was consistent with the recent promotion of large-scale HN agencies by the Ministry of Health, Labour and Welfare; recent research on a small Japanese sample also suggested that large HN agencies provided significantly more home-based EOL care [18]. The larger scale of these agencies makes it possible to support clients round-the-clock, while also providing quality staff education [19, 20]. We also found a similar association between agency size and home-based EOL care among CM agencies. These findings indicate the importance of developing large-scale home-care agencies.

The importance of organizational support was also shown by the fact that HH agencies with a policy of accepting EOL clients had more EOL case(s). Another analysis of this survey also showed that accepting EOL cases in HH agencies was associated with higher self-evaluation for EOL care collaborations among HH agencies [21]. In HH in particular, it is often necessary that the agency as a whole takes the stance of promoting EOL care before individual members of the staff can provide EOL care themselves. An organizational approach should be considered to further enhance home-based EOL care among those agencies.

The results indicated that inter-agency collaboration was also associated with the provision EOL care. Multiple findings regarding the collaboration with CM agencies strongly suggest that the care manager is actually a key professional in community-based collaborations [22]. However, this study was not sufficient to clarify the details of effective collaboration. We should further examine this aspect in future studies.

When comparing the factors related to the EOL care cases, we found that there are some differences among the 3 types of home-care agencies. In HN agencies, the number of staff and collaboration with CM agencies were significant factors; this suggests that it is important for HN agencies to provide care services that support clients around the clock, and that quality staff education and support from care managers are particularly important. Also, in CM agencies, the number of staff and collaborations with home-care nurses and home helpers were important in conducting EOL care. On the other hand, in HH agencies, organizational policies of accepting EOL clients was a significant factor. Thus, these agencies require different strategies to increase the numbers of EOL care cases and further promote home-based EOL care.

### Limitations

This study does have several limitations. The study was cross-sectional and the observed association among variables might not be causal. What’s more, the response rate of the survey was not very high, and we could not clarify the characteristics of non-responders; therefore, there might be a response bias in such a way that the responders were mainly those involved in a large number of EOL care cases or those providing high-quality EOL services. In future studies, we should evaluate the impact of the institutional factors revealed in this study on the numbers of EOL care cases. This can be done by using a larger sample, such as one from a national database.

## Conclusions

In this cross-sectional survey, we clarified and compared factors related to EOL cases among three types of home-care agencies. In HN agencies, the number of staff and the number of collaborating CM agencies were detected as being the most significant factors; in HH agencies, accepting EOL clients into the agency was a strongly significant factors; in CM agencies, the number of staff, the number of collaborating HH agencies, and whether home-care nurses and home helpers visit clients together were observed as the most significant factors. These results suggest that we need different approaches to promote home-based EOL care in each type of agency. Additionally, support from home-care organizations and the LTCI system, coupled with the effective management of community-based collaborations among professionals, is equally important in facilitating home-based EOL care.

## Abbreviations

CI: confidence interval
CM: care management
EOL: end-of-life
HH: home help
HN: home-care nursing
LTCI: long-term care insurance
OR: odds ratio
SE: standard error
